# Outcome of uterine rupture and associated factors in Yirgalem general and teaching hospital, southern Ethiopia: a cross-sectional study

**DOI:** 10.1186/s12884-020-02950-8

**Published:** 2020-04-28

**Authors:** Achamyelesh Gebretsadik, Hailemichael Hagos, Kebede Tefera

**Affiliations:** 1 School of Public Health, College of Medicine and Health Science, Hawassa University, P.O. Box 1466, Hawassa, Ethiopia; 2 School of Medicine, Department of Obstetrics and Gynecology, Hawassa University, Hawassa, Ethiopia

**Keywords:** Uterine rupture, Outcome, Ethiopia

## Abstract

**Background:**

The occurrence of uterine rupture has dropped significantly in high income countries. It continues, however, to be a major public and clinical health problem in low income countries including Ethiopia. Aim of this study was to assess management outcomes of uterine rupture and associated factors in Yirgalem General and Teaching Hospital in South Ethiopia.

**Methods:**

Institution-based cross-sectional study was conducted to examine medical records of women with uterine rupture between January 1, 2012, and Decem”ber 31, 2017. Data were collected based on a checklist. Descriptive statistics and logistic regression analyses were performed.

**Results:**

Incidence of uterine rupture was 345 in 13,500 live births (25.5 in 1000 live births) in the study period. Of these, 331 cases were included. Poor maternal outcome occurred in 224 (67.7%) women. There were 13 (3.7%) maternal deaths and 320 (96.7%) stillbirths. Wound site infection (131; 39.6%) and anemia (129; 39%) were the most common post-operative complications. Prolonged duration of labor (more than 24 h) (adjusted odds ratio (aOR) 3.6; 95% CI 1.7–7.4), women with sepsis on admission (aOR 2.9; 95% CI 1.4–6.1), hemoglobin level < 7 g/dl prior to surgical intervention (aOR 4.5; 95% CI 1.1–17.8), delayed surgical intervention after hospitalization (4 h or more before surgery) (aOR 3.8; 95% CI 1.8–8), women who did not receive blood transfusion (aOR 4.0; 95% CI 2.1–7.9) and prolonged intraoperative time (aOR 5.5; 95% CI 2.8–10.8) were all factors associated with poor maternal outcome of uterine rupture.

**Conclusion:**

Poor maternal outcome of uterine rupture was high in the study area as compared to other studies. Proper management of anemia, prompt surgical treatment, proper labor progress monitoring, surgical skills, improved infection prevention, maximizing blood transfusion availability and improving the quality of maternal healthcare all play a significant role in reducing uterine rupture and enhancing the chance of good outcomes.

## Background

Uterine rupture may occur in an intact or scarred gravid uterus from previous caesarean section or other uterine surgeries [1]. It results in significant maternal and perinatal morbidity and mortality [2].

In 2015, worldwide 303,000 women died every year due to complications of pregnancy and childbirth [3]. Uterine rupture accounts for 8% of all maternal deaths with the majority of cases occurring in low-income countries, bearing over 90% of the burden of all maternal deaths [4, 5]. Ethiopia continues to have one of the highest maternal and perinatal mortality in sub-Saharan Africa [6–8].

According to the World Health Organization (WHO), a systematic review of maternal mortality and morbidity revealed the incidence of uterine rupture in high income countries to be 0.92% [4]. However, in low-income countries, a wider variation of uterine rupture prevalence was observed with 1.9% in Central Africa, 18% in Burkina Faso and 28% in Ethiopia [9, 10].

Risk factors for uterine rupture are: oxytocin induction or augmentation, trial of labour after caesarean birth, poor ANC follow up, prolonged labour, home birth, teenage pregnancy, grand multigravidity, low socioeconomic status, poor infrastructure and poor referral system [11–13].

Uterine rupture can happen in health facilities due to delays in seeking appropriate care at the onset of labour, delays in referral system and delays of intervention due to lack of skilled human resources and negligence of health professionals that ultimately result in poor outcome [12].

The high perinatal and maternal mortality associated with uterine rupture is occurring largely as a result of improper and delayed management [14–19]. Overall, few studies are available nationally in Ethiopia depicting the outcome of uterine rupture. Therefore, aim of this study was to assess outcome of uterine rupture and its associated factors.

## Methods

### Study setting and design

The study was conducted in Yirgalem General and Teaching Hospital (YGTH) in Yirgalem town, Sidama zone, which is located 318 Km south of Addis Ababa, the capital of Ethiopia and 45 km south of Hawassa, the capital of Southern Nation Nationalities People Region (SNNPR). Catchment area includes 4.2 million people in the Sidama zone and surrounding area.

The hospital has seven departments, including Obstetrics and Gynecology, where laboring mothers are admitted, monitored and managed accordingly. This department comprises of 3 gynecologists, 4 general practitioners, 3 emergency surgical officers and 18 midwives. Most cases are referred by neighboring district hospitals and health centers. Total number of births during the six-year period between January 1, 2012 and December 31, 2017 was estimated to be 13,500. The hospital has the capacity to perform basic and comprehensive emergency obstetric care, including management of uterine rupture.

A facility-based cross-sectional survey was conducted through records review among women who were diagnosed with a ruptured uterus during the study period and managed at Yirgalem General and Teaching Hospital.

### Study population

All women admitted with uterine rupture were included. Obstetric and operative theatre records were reviewed. A total of 345 patients’ cards were identified and all complete medical records with a diagnosis of uterine rupture were assessed. Patients with incomplete medical records (missing data or records; *n* = 10) or patients whose records were lost (*n* = 4) were excluded from the study. Thus 331 women could be included.

### Data collection

A checklist was developed to collect data which were then pre-tested. This included socio-demographic data, obstetric history and outcome of uterine rupture. Checklists were prepared in English and data were extracted from the patients’ medical records, registration books and available operation theatre records by four data collectors and one supervisor. All data collectors and supervisor were selected using their previous experience in data collection, and based on their profession (being either a nurse or midwife). Before data collection, two days of training were provided by the investigators to familiarize the teams with the study objectives and tools.

Quality of the data was assured by a properly designed data collection format and managed by appropriate supervision of data collectors. The checklists were pretested at YGTH from 2011 medical records for those women with uterine rupture management. Correctness of entered data was evaluated by re-reviewing the patients’ medical records by the supervisor.

### Data analysis

Data were checked for errors, coded, entered, cleaned, and analyzed by SPSS version 20.0 statistical package. Descriptive statistics (mean ± standard deviation, frequencies and proportions) were used to summarize socio-demographic characteristics and logistic regression was conducted to assess risk factors of uterine rupture (95% CI and *P*-value < 0.05 as level of significance). Variables with a *p*-value ≤0.25 or unadjusted odds ratios which showed a significant association were entered into multivariate binary logistic regression. Goodness of fit was assessed by the Hosmer-Lemeshow test when not significant (*p*-value > 0.05) and omnibus test which showed significance (*P*-values < 0.001).

### Operational definitions

#### Uterine rupture

Uterine rupture with complete disruption of the uterine wall only.

#### Outcome of uterine rupture

Maternal condition post uterine rupture, including survival, complications like wound site infection, anemia, pelvic abscess, pneumonia, urinary tract infection, vesico-vaginal fistula or maternal death [13].

#### Stillbirth

Expulsion of a dead fetus weighing at least 1000 g [5].

#### Good maternal outcome

Those women with uterine rupture who are discharged without any complication [20].

#### Poor maternal outcome

Women with uterine rupture who died or developed complications [14].

#### Pelvic abscess

A collection of infected fluid in the pouch of Douglas, Fallopian tube, ovary, or parametric tissues.

## Results

### Socio -demographic characteristics

From 2012 up to the end of 2017 345 cases of ruptured uterus were identified. Out of these, only 331 had complete medical records available for analysis.

Mean age was 31.47 (SD ±5. 65) years with a range of 18–40 years. Rural dwellers accounted for 279 (84.3%) and urban dwellers for 52 (15.7%). (Table 1).

**Table 1:** Socio-demographic and obstetric profiles of women who were managed for uterine rupture in Yirgalem general and teaching hospital, Sidama Zone, southern Ethiopia, 2012–2018

Variables (*N* = 331)	Category	Frequency N (%)
Age (Years)	< 20	14 (4.2%)
	20–24	19 (5.7%)
	25–29	81 (24.5%)
	30–34	93 (28.1%)
	= > 35	124 (37.5%)
Place of residence	Rural	279 (84.3%)
	Urban	52 (15.7%)
Gestational age in weeks	37–42	258 (77.9%)
	> 42	73 (22.1%)
Parity	Primipara	29 (8.8%)
	2–4	170 (51.4%)
	> = 5	132 (39.9%)
Duration of labor	< 12 h	24 (7.3%)
	12-24 h	151 (45.6%)
	> 24 h	156 (47.1%)
Mother came by referral	Yes	154 (46.5%)
	No	177 (53.5%)
Duration of hospital stay before operation in hrs.	< 4 h	172 (51.9%)
	> = 4 h	159 (48.1%)
ANC visits	Yes	134 (40.5%)
	No	197 (59.5%)

Parity ranged from 1 to 11 with a mean of 4 (SD ±2.75). The big majority 302 (91.3%) were multiparous and grandmultiparous women with only 29 (8.8%) primiparous women. The majority (197; 59.5%) did not attend ANC. Gestational age on admission ranged between 37 and 42 weeks in 258 (77.9%) and 73 (22.1%) were > 42 weeks of gestation. (Table 1).

### Clinical features and causes

Most common complaints were abdominal pain (301; 90.9%), followed by vaginal bleeding (280; 84.6%) and cessation of uterine contractions (265; 80.1%). Intrauterine fetal death, easily palpable fetal parts and shock were the most frequent findings on admission, while sepsis and anemia was already present in almost half of the women. (Table 2).

**Table 2:** Clinical features and Causes of uterine rupture in Yirgalem general and teaching hospital, Sidama Zone, southern Ethiopia, 2012–2018

Variables (*N* = 331).	Category	Frequency N (%)
Clinical features	Abdominal pain	301 (90.9%)
	Vaginal bleeding	280 (84.6%)
	Cessation of uterine contraction	265 (80.1%)
	Absent fetal heartbeat	320 (96.7%)
Physical findings	Easily palpable fetal parts	292 (88.2%)
	Shock	248 (74.9%)
	Sepsis	149 (45%)
	Cephalopelvic Disproportion	167 (50.5%)
	Malpresentation/malposition	140 (42.3%)
Cause of uterine rupture	Previous uterine scar	62 (18.7%)
	Instrumental delivery	23 (6.9%)
	Augmentation and Induction	23 (6.9%)
	Destructive delivery	5 (1.5%)

The most common cause of uterine rupture was cephalo-pelvic disproportion (167; 50.5%) followed by malpresentation (140; 42.3%) and previous uterine scar (62; 18.7%). (Table 2).

**Fig. 1:**
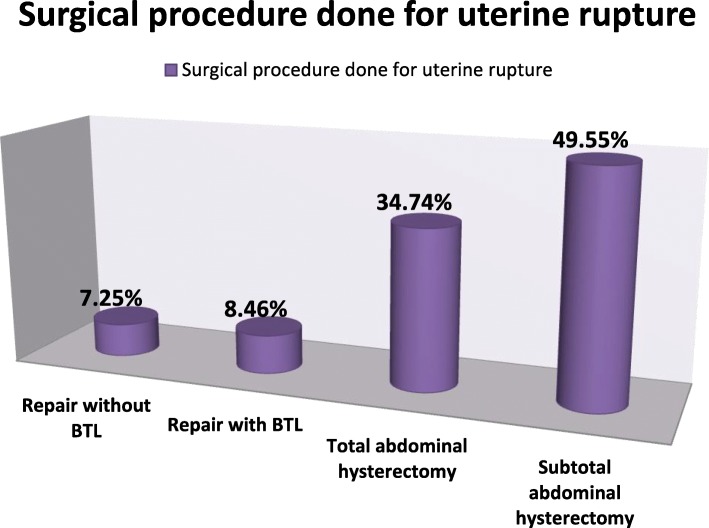
Surgical procedures for uterine rupture in Yirgalem general and teaching hospital, Sidama Zone, southern Ethiopia, 2018

Incidence of uterine rupture in YGTH was 25.5/1000 live births. A complete uterine rupture occurred in 285 (86.1%) women. The common site of rupture was anterior and lower segment in 266 (80.4%) followed by left lateral rupture in 33 (10%) women. There were 12 (3.6%) women with bladder rupture. Surgical procedures took one to four hours (mean = 1.94; SD ±0.89). About half of the women (167; 50.5%) did not receive blood transfusion. Subtotal hysterectomy was performed in almost half (164/331; 49.6%) and total hysterectomy in 115/331 (34.7%). (Fig. 1).

The most common postoperative complications were wound site infection 131 (39.6%), anemia (129; 39%) and pelvic abscess (112; 33.8%). Only 96 (29%) women had no complications. (Table 3).

**Table 3:** Post-operative complications

Variable	Category	Frequency (*N* = 331)
Complication	Wound site infection	131 (39.6%)
	Anemia	129 **(**39%)
	Pelvic abscess	112 (33.8%)
	Pneumonia	47 (14.2%)
	Urinary tract infection	36 (10.9%)
	Vesico-vaginal fistula	7 (2.1%)
	No complication	96 (29%)

Multiple responses possible

### Maternal and perinatal outcome

**Fig. 2:**
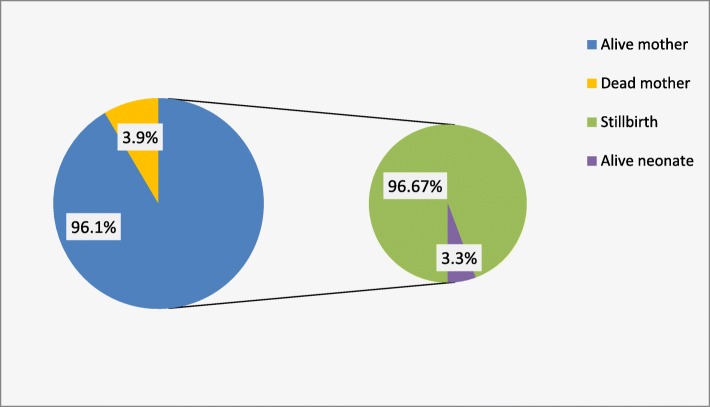
Maternal and Perinatal outcome of uterine rupture in Yirgalem General & Teaching Hospital from 2012 to 2018

There were 13 (3.7%) maternal deaths, with eight women dying from multiple organ failure secondary to septic shock and five from cardiorespiratory arrest secondary to hypovolemic shock. All deaths occurred among women who did not attend ANC. Regarding the overall prognosis, 224 (67.7%) had a poor maternal outcome, and all women had a hospital stay of at least eight days. There were 320 (96.7%) stillbirths, of which 10 (3.3%) were macerated. (Fig. 2).

### Factors associated with poor outcome

Binary logistic analysis indicated rural residence, women without attending ANC, prolonged labor (> 24 h), delay of surgical intervention (> 4 h after hospitalization), prolonged surgical procedures (> 2 h), sepsis (septic shock), complete uterine rupture, not receiving blood transfusion, and hemoglobin level < 7 mg/dl on admission to be significantly associated with poor maternal outcome.

In a multivariate logistic regression analysis, hemoglobin level < 7 mg/dl on admission (aOR 4.5; 95% CI 1.1,17.8), delay of surgical intervention > 4 h after hospitalization (aOR 3.8; 95% CI 1.8, 8), prolonged labor > 24 h (aOR 3.6; 95% CI 1.7, 7.4), maternal sepsis on admission (aOR 2.9; 95% CI 1.4, 6.1), no blood transfusion (aOR 4; 95% CI 2.1, 7.9) and intraoperative duration > 2 h (aOR 5.5; 95% CI 2.8, 10.8) were all associated with poor maternal outcome. (Table 4).

**Table 4:** Factors associated with poor maternal outcome of uterine rupture

Variables	Poor outcome of uterine rupture		cOR(95%CI)	aOR(95%CI)
	Yes (%)	No (%)		
Residence				
Urban	22 (42.3%)	30 (57.7%)	1	1
Rural	202 (72.4%)	77 (27.6%)	3.57 (1.9–6.5)*	2 (0.83–4.7)
Time from admission to surgery				
< 4 h	82 (47.7%)	90 (52.3%)	1	1
= > 4 h	142 (89.3%)	17 (10.7%)	9.2 (5–16.5)*	3.8 (1.8–8)*
Type of uterine rupture				
Complete	208 (73%)	77 (27%)	5 (2.6–9.8)*	2.2 (0.96–5)
Incomplete	16 (34.8%)	30 (65.2%)	1	1
Hemoglobin level				
< 7 g/dl	57 (93.4%)	4 (6.6%)	8.7 (3.1–24.9)*	4.5 (1.1–17.8)*
= > 7 g/dl	167 (61.9%)	103 (38.1%)	1	1
ANC				
Yes	63 (47%)	71 (53%)	1	1
No	161 (81.7%)	36 (18.3)	5 (3–8.3) *	1.6(.78–3.3)
Sepsis on admission				
Yes	130 (87.2%)	19 (12.8%)	6.4 (3.6–11.2)*	2.9 (1.4–6.1) *
No	94 (51.6%)	88 (48.4%)	1	1
Duration of lab or in hr.				
> 24 h.	131 (84%)	25 (16%)	4.6 (2.7–7.7) *	3.56 (1.7–7.4) *
< 24 h.	93 (53.1%)	82 (46.9%)	1	1
Blood transfused				
Yes	88 (53.7%)	76 (46.3%)	1	1
No	136 (81.4%)	31 (18.6%)	3.8 (2.3–6.2)*	4 (2.1–7.9) *
Surgery length				
= > 2 h	173 (84.8%)	31 (15.2%)	8.3 (4.9–14) *	5.5 (2.8–10.8)*
< 2 h	51 (40.2%)	76 (59.8%)	1	1

Note: *p < 0.05

## Discussion

Poor maternal and perinatal outcome of uterine rupture was high in Southern Ethiopia (67.7%) and remains one of the most serious obstetric complications. Most women did not attend ANC. Duration of hospital stay before operation for ≥4 h, hemoglobin level < 7 g/dl before operation, prolonged labor, sepsis, prolonged duration of the surgical intervention and not available blood transfusion were statistically significant associated factors.

The incidence of uterine rupture of 25.5/1000 live births was higher than in studies in Uganda and India [21, 22]. Overall, 67.7% of cases resulted in poor maternal outcome. This was much higher than in studies in high income countries (15%) [19].

This variation could be due to delay of health-seeking behavior, poor quality of health care services, shortage of blood and blood products for transfusion, prolonged operating time due to lack of professional technical skills and severity of uterine rupture.

During this period, case fatality rate was 3.7% after emergency hysterectomy due to heavy vaginal bleeding or sepsis, which led to hypovolemic or septic shock. Considering lack of blood and delayed treatment, many women developed sepsis and multiorgan failure. These findings are comparable to a study in Debremarkos Hospital [20], but lower than another one in Shashemene Hospital [23].

This study showed that women with hemoglobin levels < 7 g/dl prior to surgical intervention was significantly associated with poor maternal outcome. This is partially explained by the fact that anemic women have decreased oxygen carrying capacity with subsequent delayed wound healing and increased risk for infection. This is consistent with studies performed in Suhul General Hospital at Tigrai and Debremarikos [7, 20]. Improving hemoglobin levels during pregnancy is an important part of ANC. A striking feature in our study was a gestational age > 42 weeks in 22.1%. This is likely a result from late ANC booking and calls for early initiation of ANC in the first trimester.

Prolonged labor/obstructed labor was significantly associated with poor outcomes. Appropriate use of partograph and monitoring progress of labor as well as prompt intervention within 4 h after admission is necessary to prevent both fetal and maternal morbidity and mortality [24]. These findings are consistent with studies in Suhul General Hospital at Tigrai, Mizantepi Hospital, and Nnamdi Azikiwe University Teaching Hospital Nnewi, Southeast Nigeria [7, 14, 15].

A statistically significant association was also noted with a prolonged surgical procedure of > 2 h duration. Increased blood loss and postoperative anemia, increased risks of infection, prolonged exposure to anesthesia drugs which predispose to aspiration pneumonia and other drug side effects are determinants of poor maternal outcome. In addition, professionals may lack experience and skills leading to prolonged surgical procedures and complications. This was reported also in a study from the University of Pakistan teaching hospital [17].

Not receiving blood transfusion was statistically associated with poor maternal outcome in uterine rupture. This is likely explained by tremendous vaginal bleeding which is common in uterine rupture. Resuscitation with blood transfusion may prevent development of serious complications. This is consistent with a study from the Medical College of India [18].

A woman with sepsis on admission was more likely to develop complications and multiple end organ failure resulting from infection-related hypotension and unbalanced inflammatory response [1, 13]. A similar finding was reported in Tanzania [25]. Women with sepsis were at high risk of developing multiorgan failure, due to aggressive immune-mediated organ injury leading to severe septic shock and death [1, 13].

### Limitations of the study

All data were collected from secondary sources (medical records) and not supported by direct observation and interviewing of the women. Another challenge was poor documentation and inconsistencies of medical records. Results of our facility-based study may not be generalizable for the community at large.

## Conclusion

Poor maternal outcome among women with uterine rupture managed in Yirgalem General and Teaching Hospital was high compared to other studies. Delayed surgical intervention, hemoglobin < 7 g/dl and sepsis before surgical intervention, prolonged duration of labor, prolonged duration of surgery and lack of blood transfusion were all factors associated with poor outcome. Therefore, the government and other responsible stakeholders should focus attention on proper and immediate identification of risk factors and initiation of timely management. Capacity building through training of healthcare professionals, assessing barriers to blood transfusion, and improving access to ANC and standard care during pregnancy, including early calculation of gestational age, and childbirth are crucial to prevent and better manage uterine rupture. Proper labor follow-up and infection prevention and control are also important for better outcome. Maximizing the quality of maternal health care is crucial for reducing maternal and perinatal morbidity and mortality.

## Data Availability

The datasets generated and/or analyzed during the current study available from the corresponding author on reasonable request.

## Abbreviations

aOR: Adjusted Odds Ratio
ANC: Antenatal Care
cOR: Crude Odds Ratio
GA: Gestational age
SNNPR: Southern Nation Nationalities People Region
YGTH: Yirgalem General and Teaching Hospital
